# Measuring intersecting stigma among key populations living with HIV: implementing the people living with HIV Stigma Index 2.0

**DOI:** 10.1002/jia2.25131

**Published:** 2018-07-22

**Authors:** Barbara A Friedland, Laurel Sprague, Laura Nyblade, Stefan D Baral, Julie Pulerwitz, Ann Gottert, Ugo Amanyeiwe, Alison Cheng, Christoforos Mallouris, Florence Anam, Aasha Jackson, Scott Geibel

**Affiliations:** ^1^ Project SOAR Population Council New York NY USA; ^2^ Global Network of People Living with HIV (GNP+) Amsterdam the Netherlands; ^3^ Division of Global Health RTI International Washington DC USA; ^4^ Johns Hopkins Bloomberg School of Public Health Baltimore MD USA; ^5^ Project SOAR Population Council Washington DC USA; ^6^ U.S. Agency for International Development (USAID) Washington DC USA; ^7^ Community Mobilization Division UNAIDS Geneva Switzerland; ^8^ International Committee of Women Living with HIV (ICW) Nairobi Kenya

**Keywords:** HIV, stigma, key population, intersectionality, Stigma Index, PLHIV

Addressing stigma affecting people living with HIV (PLHIV) is a global priority 1. Stigma, defined as “the co‐occurrence of labeling, stereotyping, separation, status loss, and discrimination in a context in which power is exercised” 2, has a negative impact on the health of PLHIV, and contributes to psychosocial stress, coercion and violence, job loss, and social exclusion 3. Among PLHIV, gay men and other men who have sex with other men (MSM), transgender individuals, sex workers, and people who use drugs (PWUD) – often referred to as key populations – are situated at the intersection of HIV‐related stigma and prejudice against their identities, occupations, or behaviours, often exacerbating their experiences of stigma and discrimination 4, 5. To support the health and rights of key populations living with HIV, it is important to understand their experiences of stigma, as well as to explore the intersectionality between HIV, key population status, and other marginalized group memberships, such as ethnic/and racial minorities, prisoners, migrants, and persons with disabilities. Yet to date, the psychometric study of intersecting patterns of stigma has been limited.

The PLHIV Stigma Index (Stigma Index) provides evidence on stigma and discrimination that has been essential for informing HIV policy, PLHIV rights advocacy efforts, and stigma‐reduction interventions 6, 7, 8, 9. Developed by the Global Network of PLHIV (GNP+), the International Community of Women Living with HIV (ICW), the International Planned Parenthood Federation (IPPF), and UNAIDS 10 the Stigma Index is a research tool by which PLHIV capture data on their experiences of stigma and discrimination. As of November 2017, more than 100,000 PLHIV had been interviewed in over 50 languages by 2000 trained PLHIV interviewers.

In 2016, the Population Council's Project SOAR and the Stigma Index partners (UNAIDS, GNP+, ICW), with PEPFAR funding from the Office of HIV/AIDS Research of the United States Agency for International Development (USAID), undertook a process to update the Stigma Index to more fully capture the experiences of key populations living with HIV, as well as to respond to changes in global treatment guidelines, and to better understand the persistent barriers to HIV testing and treatment. Throughout the consultative, iterative process of revising the Stigma Index questionnaire, multiple stakeholders, including PLHIV networks and advocates, were asked for their input. A common recommendation was to enable respondents to share experiences of both HIV‐related and key population‐related stigma. Based on lessons learned from implementation in over 90 countries, stakeholders made a number of specific suggestions that were incorporated into the updated Stigma Index, including: adding a separate section with specific questions to measure stigma experienced by key populations; ensuring background demographic questions would enable analysis of stigma and discrimination by sub‐population; integrating a validated two‐part gender identity question into the demographics section; separating gender identity from sexual orientation in questions and responses; utilizing existing surveys and scales for measuring stigma and discrimination in key populations 11, 12, 13; and narrowing the timeframe to the past 12 months to better capture changes over time.

The updated survey – the Stigma Index 2.0 – was pilot‐tested by the National Forum of PLHIV Networks in Uganda (NAFOPHANU) in Kampala, Uganda; by Metabiota, in collaboration with ReCAP+ in Douala and Yaoundé, Cameroon; and by Enda Santé, in collaboration with RNP+ in Dakar and Ziguinchor, Senegal. PLHIV were recruited from PLHIV networks, community‐based organizations serving key populations, ART clinics, and through snowball sampling. Data were collected on tablet computers (Cameroon and Senegal) or mobile phones (Uganda) in multiple languages. The pilot study protocol was approved by local ethics committees in each country, and by the Institutional Review Boards of the Johns Hopkins School of Public Health (Baltimore, MD, USA) and the Population Council (New York, NY, USA).

Respondents were classified as “key populations” based on two items in the demographics section: 1) discordant responses to the two‐part gender identity question (i.e. born “male,” currently identify as “female”) or 2) a “yes” response to group identity (MSM, lesbian, transgender, sex‐worker, PWUD); or a “yes” response to any of five questions about behaviours in the new key population section (e.g – to men only, “have you ever had sex with another man”).

In 2017, 1204 PLHIV completed the survey, approximately 40% of whom identified as a member of at least one key population group (40% Cameroon; 34% Senegal; 43% Uganda). Of the total sample, 18% of respondents identified as sex workers; 10% as gay or MSM; 5% as transgender (men and women), 3% as PWUDs, and <1% as lesbian 14. A substantial proportion of respondents reported having ever experienced at least one of 11 forms of HIV‐related stigma, such as exclusion from family, social, religious activities; verbal or physical harassment; or blackmail. In Senegal, the country with the lowest reports of HIV‐related stigma, 33% of key population members experienced at least one form of stigma compared to 18% of other respondents. By contrast, in Cameroon and Uganda, where prevalence of stigma was higher, overall, experienced stigma among key populations and the other respondents was similar (76% vs. 75%, Cameroon; 53% vs. 51%, Uganda) 14.

The new key population section indicated higher levels of stigma and violence in Cameroon than the other two countries. Among MSM living with HIV, the most commonly reported type of stigma was verbal harassment, which was reported by nearly twice as many MSM in Cameroon (83%) as in Senegal (44%) or Uganda (32%). Among transgender women living with HIV, “unjust remarks by family” was reported by nearly three times as many respondents in Cameroon (89%) than in Senegal (36%) or Uganda (30%). Differences by country in abuses experienced by sex workers living with HIV was less pronounced; forced sex was reported by 52%, 41% and 32% of respondents from Cameroon, Senegal, and Uganda, respectively.

The widespread adoption of the original PLHIV Stigma Index 6, 10 and use of results for global 15, 16, 17 and national 18, 19 reporting, as well as for empowerment and advocacy by and for people living with HIV 20, 21, demonstrated the demand for a tool to monitor stigma and discrimination worldwide, and to support and develop leadership among people living with HIV. With its expanded scope and agility, the Stigma Index 2.0 creates opportunities for countries and communities to implement a tool that yields evidence‐based data including measurement of the intersectionality of stigma affecting key populations living with HIV to more effectively implement stigma mitigation interventions as part of human rights‐affirming HIV responses. Furthermore, with the emphasis on experiences of stigma and discrimination in the past 12 months, the Stigma Index 2.0 can be used to monitor progress and challenges over time. Given the potential impact on the response to the AIDS epidemic, proactive efforts to support PLHIV networks and in‐country partners to implement the Stigma Index 2.0 and dissemination of results should be encouraged.

## Authors’ contributions

BF drafted the manuscript. BF, JP, SB, LN and AG conceptualized key messages. LS, LN, SB, UA, AC, CM, JP and SG edited the manuscript. CM, JP, SB, AG, LN, SG, BF, FA, UA, AC, AJ and LS contributed to the Stigma Index revisions and interpreted results.

## Funding

Support for the Stigma Index update through the Population Council's Project SOAR (Cooperative Agreement AID‐OAA‐A‐14‐00060) was made possible by the generous support of the American people through the President's Emergency Plan for AIDS Relief (PEFPAR) and the U.S. Agency for International Development (USAID).

## Disclaimer

The contents of this viewpoint are the sole responsibility of the authors and do not necessarily reflect the views of the U.S. President's Emergency Plan for AIDS Relief, the U.S. Agency for International Development or the U.S. Government.

## Competing interests

The authors have no conflicts of interest to declare.
